# Supplementary material for: Features of patients that died for COVID-19 in a hospital in the south of Mexico: A observational cohort study
Source: PLoS One. 2021 Feb 19;16(2):e0245394. doi: 10.1371/journal.pone.0245394 (PMC7894952; doi:10.1371/journal.pone.0245394)
Supplement: Attachment — Submitted filename: Response to Reviewers.docx [file pone.0245394.s001.docx]

**miércoles, diciembre 02, 2020**

**Francesco Di Gennaro**

**Academic Editor**

**PLOS ONE**

Thank you very much for giving us the opportunity of revising our manuscript. Also, we greatly appreciate the time and effort the reviewers spent in our manuscript. We would like to re-submit the paper entitled: “Features of patients who died of COVID-19 in a Hospital in the south of Mexico: A observational cohort study”, (Ref: PONE-D-20-29232); by Jesús Arturo Ruíz-Quiñonez, Crystell Guadalupe Guzmán-Priego, German Alberto Nolasco-Rosales, Carlos Alfonso Tovilla-Zarate, Oscar Israel Flores-Barrientos, Víctor Narváez-Osorio, Guadalupe del Carmen Baeza-Flores, Thelma Beatriz Gonzalez-Castro, Carlos Ramón López-Brito, Carlos Alberto Denis-Garcia, Agustín Pérez-García, Isela Esther Juárez-Rojop, in PLOS ONE.

My colleagues and I believe this revision has been markedly improved. The answers to your remarks together with point to point clarifications are listed below; these include the changes asked and kindly suggested by the referee. The authors are indebted to the referee for improving the quality of the present article with their remarks. We have carefully considered the reviewer’s comments and Editor´s remarks, we have answered them all and performed each of the changes proposed in your revision as follows:

**Editor and Reviewer Comments:**

Journal Requirements:

When submitting your revision, we need you to address these additional requirements.

**1. Please ensure that your manuscript meets PLOS ONE's style requirements, including those for file naming.** The PLOS ONE style templates can be found at https://journals.plos.org/plosone/s/file?id=wjVg/PLOSOne_formatting_sample_main_body.pdf and

https://journals.plos.org/plosone/s/file?id=ba62/PLOSOne_formatting_sample_title_authors_affiliations.pdf

We do.

**2. Please provide additional details regarding participant consent. In the ethics statement in the Methods and online submission information, please ensure that you have specified (i) whether consent was informed and (ii) what type you obtained (for instance, written or verbal, and if verbal, how it was documented and witnessed).**

If your study included minors, state whether you obtained consent from parents or guardians.

If the need for consent was waived by the ethics committee, please include this information.

If you are reporting a retrospective study of medical records or archived samples, please ensure that you have discussed whether all data were fully anonymized before you accessed them and/or whether the IRB or ethics committee waived the requirement for informed consent. If patients provided informed written consent to have data from their medical records used in research, please include this information.

We included the information of ethics committee approval.

**3. Please ensure that you include a title page within your main document. You should list all authors and all affiliations as per our author instructions and clearly indicate the corresponding author.**

We made the changes.

**4. PLOS requires an ORCID iD for the corresponding author in Editorial Manager on papers submitted after December 6th, 2016. Please ensure that you have an ORCID iD and that it is validated in Editorial Manager. To do this, go to ‘Update my Information’ (in the upper left-hand corner of the main menu), and click on the Fetch/Validate link next to the ORCID field. This will take you to the ORCID site and allow you to create a new iD or authenticate a pre-existing iD in Editorial Manager. Please see the following video for instructions on linking an ORCID iD to your Editorial Manager account:** [**https://www.youtube.com/watch?v=_xcclfuvtxQ**](https://www.youtube.com/watch?v=_xcclfuvtxQ)

We included the ORCID.

**5. Please amend either the abstract on the online submission form (via Edit Submission) or the abstract in the manuscript so that they are identical.**

**Additional Editor Comments:**

Dear Authors follow reviewer suggestion to improve your article

Reviewers' comments:

Reviewer's Responses to Questions

**Comments to the Author**

**Is the manuscript technically sound, and do the data support the conclusions? The manuscript must describe a technically sound piece of scientific research with data that supports the conclusions. Experiments must have been conducted rigorously, with appropriate controls, replication, and sample sizes. The conclusions must be drawn appropriately based on the data presented.**

Reviewer #1: Partly

Reviewer #2: Yes

Reviewer #3: Yes

**2. Has the statistical analysis been performed appropriately and rigorously?**

Reviewer #1: No

Reviewer #2: Yes

Reviewer #3: Yes

**3. Have the authors made all data underlying the findings in their manuscript fully available?**

**The PLOS Data policy requires authors to make all data underlying the findings described in their manuscript fully available without restriction, with rare exception (please refer to the Data Availability Statement in the manuscript PDF file). The data should be provided as part of the manuscript or its supporting information, or deposited to a public repository. For example, in addition to summary statistics, the data points behind means, medians and variance measures should be available. If there are restrictions on publicly sharing data—e.g. participant privacy or use of data from a third party—those must be specified.**

Reviewer #1: No

Reviewer #2: Yes

Reviewer #3: Yes

**4. Is the manuscript presented in an intelligible fashion and written in standard English?**

**PLOS ONE does not copyedit accepted manuscripts, so the language in submitted articles must be clear, correct, and unambiguous. Any typographical or grammatical errors should be corrected at revision, so please note any specific errors here.**

Reviewer #1: No

Reviewer #2: Yes

Reviewer #3: Yes

**5. Review Comments to the Author**

**Please use the space provided to explain your answers to the questions above. You may also include additional comments for the author, including concerns about dual publication, research ethics, or publication ethics. (Please upload your review as an attachment if it exceeds 20,000 characters)**

**Reviewer #1: I thank the authors for the opportunity to read their interesting work presenting findings of deceased COVID-19 patients. However, I have some concerns regarding this study**

**1. English is poor please have a native speaker read through your text**

Done

**2. COVID-19 as per death certificate does not necessarily represent cause of death or associated secondary complications that would be of interest to the reader. Similarly, information on comorbidies is poor. How many of these patients were immunosuppressed? What drugs were they receiving? Etc**

We modified this information (Page 6, line 110-111; page 8, line 145-150)

*Change in the manuscript:*

“All patients included in this study died of cardiorespiratory arrest, secondary to acute respiratory failure. […] Other less frequent comorbidities observed in individuals who died by COVID-19 are depicted in Table 3. There was one individual with HIV and other two with cancer; the three of them were immunosuppressed. It is important to mention that these individuals were also receiving the usual treatment for their underlying disease while they were treated in hospital for COVID-19.

**3. Discussion of your findings in line with different epidemiological studies in other parts of the world is necessary. At the moment is just a report of your results.**

We include information in Discussion section (Page 11; page 12, line 213, 214, 218-222).

*Change in the manuscript:*

Fever and cough had been reported as the most prevalent symptoms in case series from Europe, China, Mexico and in severe cases. On the other hand, the third most prevalent symptom varied widely; for instance, a review found dyspnea in 53 – 80% of cases, while a series of fatal cases in Wuhan found dyspnea in 70.6%. Nonetheless, in clinical series and meta-analysis involving non-lethal cases in Asia, Europe, and North America, the third most prevalent symptom was myalgias or fatigue (31-67%). […] In a follow-up study of 20,133 individuals with COVID-19 in United Kingdom, the most frequent comorbidities were chronic cardiac disease (31%), uncomplicated diabetes (21%), non-asthmatic chronic pulmonary disease (18%), and chronic kidney disease (16%); while only 23% reported no major comorbidities. On the other hand, a report in Northeast Brazil showed that the mortality of COVID-19 was enhanced by old age, neurological diseases, pneumopathies and cardiovascular diseases. […] Nonetheless, hypertension has a much higher prevalence compared to diabetes in reports from USA, Italy and China. […] On the other hand, in a study using a UK database, hypertension was associated with an increased risk of COVID-19 (HR 1.09) but there was no association when the risk was adjusted to age, gender, diabetes and obesity. In addition, diabetes and obesity were associated with an increased risk of death (HR 1.90 and 1.92, respectively).

**Reviewer #2: The manuscript is technical sound and statistical analysis performed appropriately. I feel the results should have also been expressed in a table format. The paper meets the PLOS ONE criteria for publication.**

We modified this point, we included Table 2, 3 and 4 (page 7, line 132; page 9, line 156 and 164).

**Reviewer #3: The manuscript is good even in Mexico there is not enough data published on PubMed with by mexicans authors. We know the lack of the real numbers of people affected by covid-19. I think, this information could be different if we compare with the North of Mexico or even with Mexico City.**

**Response 6.** We included evidence about Mexico data (page 10, line 180-182; page 11, line 203-206)

*Change in the manuscript:*

Similar findings had been observed in the state of Sonora, Northwest of Mexico, with a mean age of 56.98 years old in individuals who died by COVID-19. […] It has been reported that in Mexican individuals with COVID-19 there is a slightly higher frequency of hypertension than diabetes. Additionally, a report from Northwest Mexico indicated that diabetes and hypertension have higher risk of mortality than other comorbidities.

**6. PLOS authors have the option to publish the peer review history of their article (what does this mean?). If published, this will include your full peer review and any attached files.**

**If you choose “no”, your identity will remain anonymous but your review may still be made public.**

**Do you want your identity to be public for this peer review? For information about this choice, including consent withdrawal, please see our Privacy Policy.**

Reviewer #1: No

Reviewer #2: Yes: Victor Draman Afayo

Reviewer #3: No
